# *Yersinia enterocolitica* Infection Simulating Lymphoproliferative Disease, after Liver Transplant

**DOI:** 10.1155/2014/923058

**Published:** 2014-07-14

**Authors:** E. Jakobovich, B. Koplewitz, E. Marva, E. Granot

**Affiliations:** ^1^Kaplan Medical Center, Rehovot, Israel; ^2^Department of Medical Imaging, Hadassah Hebrew University Medical Center, Jerusalem, Israel; ^3^Central Laboratory, Ministry of Health, Jerusalem, Israel; ^4^Hebrew University, Hadassah Medical School, Jerusalem, Israel

## Abstract

We describe a 14-year-old girl, who was 13 y after liver transplantation for biliary atresia with an unremarkable postoperative course. She presented with fever of up to 40°C, extreme fatigue, malaise, anorexia, and occasional vomiting. On physical examination the only finding was splenomegaly. Lab results showed hyperglobulinemia and an elevated sedimentation rate. Liver function tests were normal except for mild elevation of *γ*GTP. Abdominal U/S and CT demonstrated an enlarged spleen with retroperitoneal and mesenteric lymph nodes enlargement. An exhaustive evaluation for infectious causes, autoimmune conditions, and malignancy was negative. A full recovery after 5 months prompted testing for self-limited infectious etiologies. *Yersinia enterocolitica* infection was diagnosed.

## 1. Introduction

In an immune suppressed host, prolonged fever in association with splenomegaly as the sole abnormal finding on physical exam may pose a difficult diagnostic challenge.

Late complications of post liver transplantation include chronic rejection, infections, autoimmune disorders, and malignancy [1]. All these can present with prolonged fever as their major symptom.

Of the infectious causes which have been described, including bacteria, viruses, fungi, and parasitic infection comprehensive reviews in immune suppressed posttransplant patients fail to include* Y. enterocolitica* among these diverse infectious etiologies [2].

We report a 14-year-old girl (13 years after liver transplantation) who presented with prolonged fever, extreme fatigue, malaise, and anorexia. On physical exam the only abnormal finding was splenomegaly. Following an extensive evaluation Yersinia infection was diagnosed.

## 2. Case Report 

A 14-year-old girl, 13 years after orthotopic liver transplantation for biliary atresia, with an unremarkable posttransplant course, on maintenance immunosuppression with tacrolimus, presented with daily fever of up to 40°C, extreme fatigue, and malaise during the preceding 3 weeks. She complained of loss of appetite and occasional vomiting, without diarrhea, abdominal pain, or arthralgia.

Physical exam was unremarkable except for an enlarged spleen (4 cm below the costal margin).

Laboratory results at that time included WBC 12,000 k/ul with a neutrophil count of 84%, hemoglobin 10.7 g% with normal indices, and platelets 298,000 k/ul, as well as ESR 53 mm/h and CRP 16 mg/L (*N* ≤ 0.6) which increased over a period of 3 weeks to 151 and 127, respectively.

Transaminases (GOT and GPT) were normal with minimal elevation of *γ*GTP to 60–80 IU/mL (normal < 45 IU/mL). Bilirubin was normal, total protein 8.9 g%, and albumin 3.5 g%.

Blood and urine cultures were negative; stool was negative for culture, parasites, and occult blood.

Serology was negative for EBV (EBV-PCR 184 copies/mL), CMV, parvovirus, Q fever, cat scratch,* Legionella*, and* Brucella*. Autoimmune markers including ANA, antismooth muscle antibody, and anti-LKM were negative.

Chest radiograph was normal; abdominal sonography demonstrated enlarged retroperitoneal and mesenteric lymph nodes (up to 1.8 cm) with an enlarged inhomogeneous spleen.

**Figure 1:**
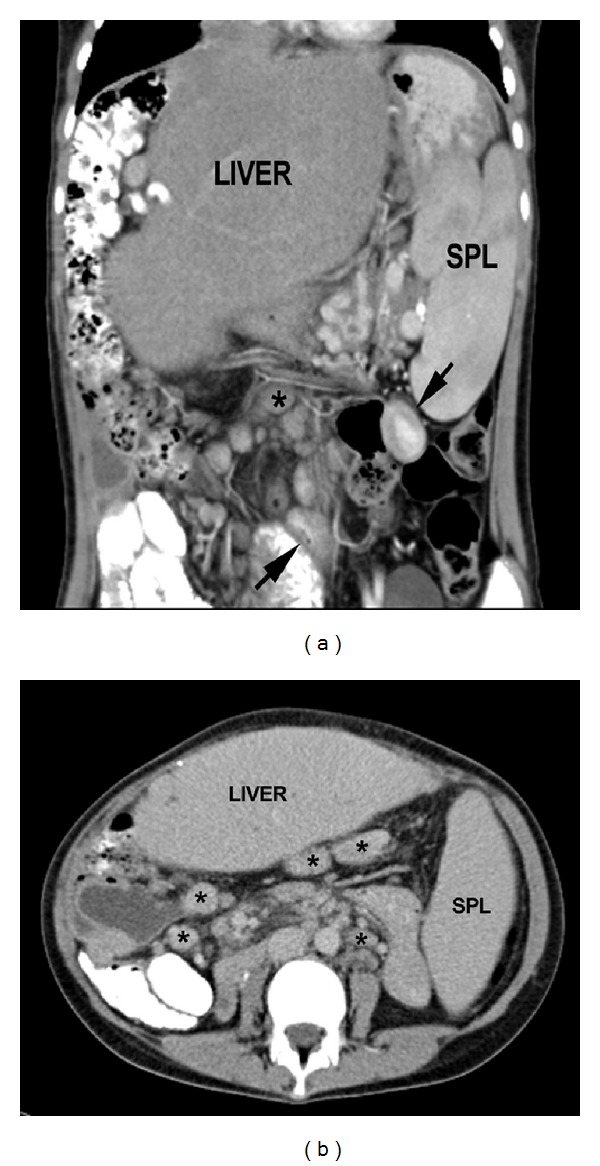
Axial (a) and coronal reformats of contrast-enhanced abdominal CT showing marked enlargement of the spleen and transplanted liver, multiple moderately enlarged mesenteric and gastrohepatic ligament lymph nodes (∗), and focal small bowel wall thickening (arrows).

On abdominal CT the enlarged lymph nodes were observed in the retroperitoneum and mesentery but none were easily accessible for needle biopsy (Figure 1).

**Figure 2:**
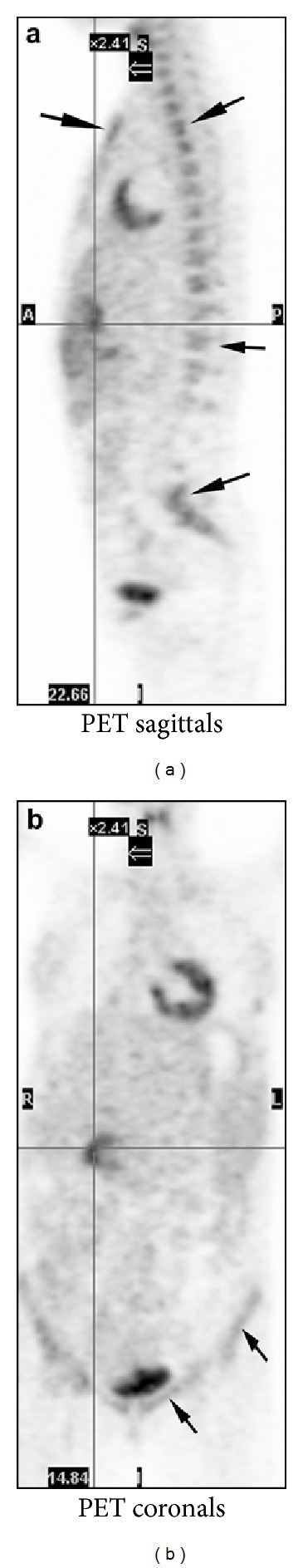
PET-CT (FDG) diffuse increased uptake (arrows) in bone marrow of the sternum and vertebral bodies (a) as well as pelvic bones (b). No pathological uptake in lymph nodes or spleen.

PET-CT (FDG) (Figure 2) showed no increased pathological activity in any of the lymph nodes and therefore no further attempt was made, at that time, to surgically remove a lymph node.

The PET-CT demonstrated diffuse increased pathological uptake in bone marrow of sternum, vertebral bodies, and pelvic bones.

Following this finding a bone marrow aspiration and biopsy were performed. The aspirate was sent for cultures and staining for* Leishmania* Donovan bodies.

The bone marrow histology was normal with normal representation of all cell lines. No pathological cells were observed and bone marrow culture and staining for* Leishmania* Donovan bodies were negative.

Over the following two months her fever gradually decreased but she continued to suffer from intermittent elevations of fever up to 38.5°C. Complaints of severe fatigue and anorexia persisted. A repeat abdominal sonography showed persistent splenomegaly and a slight decrease in lymph node size.

Over the next 3 months symptoms gradually continued to resolve, the spleen was no longer palpable, and laboratory results including ESR, CRP, hyperglobulinemia, and *γ*GTP levels gradually returned to normal.

An abdominal sonographic examination five months after initial onset of symptoms was normal with complete resolution of lymphadenopathy and splenomegaly.

Clinical recovery prompted a further attempt at a diagnostic search for an infectious, self-limiting etiology.

Serology was again sent for a multitude of infectious causes. This time testing also included* Y. enterocolitica* serology.

*Y. enterocolitica* serology was found to be positive. Antibodies of the IgG class against purified virulence factors from* Y. enterocolitica* strain 0 : 3. (ELISA kit E1 2173, Euroimmun AG) were elevated. On a blood sample obtained one month later, the antibody titer had increased twofold.

## 3. Discussion

In immune competent hosts,* Y. enterocolitica* may cause illness ranging from self-limited enteritis to infrequent life threatening infections [3]. It appears to be a common cause of diarrhea among children in Europe and Canada and has been recognized as an important seasonal gastroenteritis in certain cities in the United States. It is an infrequent infection in warm climates and is therefore rarely encountered in Israel.

In young children it usually presents as an acute diarrheal illness whereas in older children and adolescents the classic clinical presentation is that of a terminal ileitis with mesenteric lymphadenitis (pseudoappendicitis) which resolves over a 1-2-week period [4]. Septicemia caused by* Y. enterocolitica* has been rarely reported in children.

*Yersinia enterocolitica* is not commonly included in the differential diagnosis of infectious complications after liver transplantation.

In immunocompromised patients it can present as necrotizing pneumonia [5], sepsis [6], endocarditis [7], osteomyelitis [8], and bacteremia [9]. These infectious episodes are rare and are reported as isolated case reports.

Our patient had no gastrointestinal symptoms except for occasional vomiting. In retrospect the small bowel wall thickening, noted on abdominal CT coupled with the mesenteric lymphadenopathy were valuable clues to the diagnosis.

At the time of presentation the most likely diagnosis in our patient was posttransplant lymphoproliferative disorder, despite absence of evidence of EBV infection (EBV-PCR negative). The enlarged abdominal lymph nodes which were not accessible for needle biopsy were not surgically removed as they showed no pathological uptake on PET-CT and were therefore not likely to represent lymphoma.

We have found one report describing a similar clinical presentation of* Yersinia enterocolitica*. The patient was a 50-year-old woman who similarly to our patient also had prolonged fever, splenomegaly, and retroperitoneal lymph nodes. The diagnosis of* Y. enterocolitica* infection was also established only after symptoms had resolved. It is noteworthy that the patient reported was not immune suppressed [10].

Although lymphoproliferative disease remains the primary etiology in the differential diagnosis of a patient with prior liver transplantation and recent onset of prolonged fever, splenomegaly, abdominal lymphadenopathy and hyperglobulinemia, unusual infectious etiologies should also be considered. As exemplified by our patient* Y. enterocolitica* infection can simulate posttransplant lymphoproliferative disease.
